# Cross-Modality Transfer Learning from PSG to FMCW Radar for Event-Level Apnea–Hypopnea Segmentation

**DOI:** 10.3390/bioengineering13030283

**Published:** 2026-02-27

**Authors:** Saihu Lu, Peng Wang, Zhenfeng Li, Pang Wu, Xianxiang Chen, Lidong Du, Libin Jiang, Zhen Fang

**Affiliations:** 1 Aerospace Information Research Institute, Chinese Academy of Sciences (AIRCAS), Beijing 100094, China; lusaihu20@mails.ucas.ac.cn (S.L.); wangpeng01@aircas.ac.cn (P.W.); lizhenfeng@aircas.ac.cn (Z.L.); wupang@aircas.ac.cn (P.W.); chenxx@aircas.ac.cn (X.C.); lddu@mail.ie.ac.cn (L.D.); 2 School of Electronic, Electrical and Communication Engineering, University of Chinese Academy of Sciences, Beijing 100049, China; 3 Beijing Tongren Eye Center, Beijing Tongren Hospital, Capital Medical University, Beijing 100730, China

**Keywords:** transfer learning, apnea–hypopnea, FMCW radar, AHI estimation, health care

## Abstract

Sleep apnea–hypopnea syndrome (SAHS) is a common sleep-related breathing disorder associated with substantial cardiovascular and neurocognitive risks. Although polysomnography (PSG) remains the clinical gold standard for diagnosis, its cost, operational burden, and limited accessibility hinder scalable and longitudinal home monitoring. Frequency-modulated continuous-wave (FMCW) radar provides unobtrusive, non-contact respiration sensing, yet radar-based event detection is often constrained by scarce annotations and pronounced domain shifts relative to PSG signals. In this work, we propose a deep learning framework for apnea–hypopnea event detection from FMCW radar that combines a 1D U-Net segmentation backbone with multi-head self-attention (MHSA) and cross-modality transfer learning. The model was first pre-trained on a large public PSG dataset to learn transferable respiratory-event representations and then fine-tuned on a smaller clinically annotated radar respiration dataset using synchronized PSG labels. It produced per-sample event probabilities, which were further refined via temporal post-processing to generate event-level detections and apnea–hypopnea index (AHI) estimates. Experimental results demonstrate strong performance in the radar domain, achieving precision of 0.8137±0.0332, recall of 0.8369±0.0470, and an F1-score of 0.8167±0.0052. Overall, these results indicate that PSG-to-radar transfer learning enables accurate, low-cost, and non-contact sleep apnea screening, supporting scalable longitudinal monitoring in home-like settings.

## 1. Introduction

Sleep apnea–hypopnea syndrome (SAHS)—particularly obstructive sleep apnea (OSA)—is a highly prevalent sleep-related breathing disorder characterized by recurrent cessations (apnea) or reductions (hypopnea) of airflow during sleep. These respiratory events are typically accompanied by intermittent hypoxemia, fluctuations in intrathoracic pressure, and repeated arousals, resulting in fragmented sleep and impaired restorative function [1,2,3]. The clinical impact of SAHS extends beyond sleep quality. A substantial body of evidence has linked untreated SAHS to increased risks of cardiovascular morbidity (e.g., hypertension, coronary artery disease, and stroke), metabolic dysfunction, neurocognitive impairment, and reduced daytime performance, thereby posing a significant public health burden [2,4]. Because disease severity is often quantified using the apnea–hypopnea index (AHI, events/hour of sleep) and is used to guide treatment decisions and follow-up assessments, accurate diagnosis and reliable severity estimation are essential for both clinical management and long-term monitoring [5].

Despite its high prevalence and clinical consequences, SAHS remains widely underdiagnosed. Standard diagnosis relies on in-laboratory PSG, which records multi-channel physiological signals (e.g., airflow, respiratory effort, oxygen saturation, EEG, electrooculography, and electromyography) overnight. PSG provides comprehensive assessments of sleep architecture and respiratory events, with scoring standardized by the American Academy of Sleep Medicine (AASM) [5]. However, PSG is resource-intensive and burdensome, with key limitations including (i) high cost and limited sleep-lab capacity, (ii) inconvenience and discomfort from multiple sensors and wiring, and (iii) poor feasibility for repeated or longitudinal monitoring outside clinical settings [5,6]. Moreover, SAHS severity and event expression can vary night-to-night due to posture, sleep stage distribution, alcohol intake, and comorbidities, motivating unobtrusive, scalable, multi-night monitoring beyond single-night laboratory assessments [4,6].

Motivated by these needs, non-contact sensing modalities are increasingly being explored for unobtrusive sleep monitoring. Camera-based methods can estimate respiration and body movement from video, but performance may be affected by lighting, occlusion, and privacy constraints in home settings [7]. Snore sound-based approaches are also non-invasive, yet they may fail for non-snorers and can raise privacy concerns due to recording sensitive audio data [8]. In contrast, radio-frequency (RF) sensing—particularly radar—can measure subtle chest and abdominal wall motion without contact and operates reliably in darkness [9,10,11]. Radar can be deployed as a compact bedside device without wearables or adhesives, enabling continuous long-term monitoring with minimal user burden, which supports low-cost home screening and longitudinal sleep health assessment.

Despite these advantages, achieving clinically reliable apnea detection from radar remains challenging. Radar-derived respiration is highly sensitive to posture and orientation, sensor placement, motion artifacts, environmental clutter, and multipath propagation, which can distort respiratory waveforms and induce substantial inter-night variability [9,10]. In addition, mmWave and other radar modalities measure breathing indirectly via motion-related phase/amplitude variations, yielding signal morphologies and noise characteristics that differ from PSG respiratory channels. A further bottleneck is the scarcity of high-quality labeled radar datasets: accurate apnea/hypopnea annotations typically require synchronized PSG scoring under AASM criteria, which is resource-intensive and constrains cohort size [5]. Consequently, limited labels combined with pronounced PSG–radar domain shift often cause deep models trained from scratch to overfit, impeding the development of clinically robust algorithms.

Deep learning has shown strong potential for physiological time-series analysis by learning discriminative representations directly from raw or minimally processed signals. Convolutional neural networks (CNNs), recurrent networks, and transformer-based architectures have been applied to sleep staging and respiratory event detection in PSG and related biosignals. However, many radar-based studies still employ epoch-level classification (e.g., 30 s windows) rather than event-level localization. From a clinical perspective, event-level detection provides a more direct bridge to AHI estimation and severity categorization and can facilitate more interpretable post-processing (e.g., merging fragmented predictions, filtering short events) aligned with scoring rules [5].

Transfer learning provides an attractive route to mitigate data scarcity by leveraging a source domain with abundant labeled data to learn generalizable representations and then adapting the model to the target domain with limited labels [12]. In the context of sleep apnea–hypopnea detection, a large PSG dataset named The Human Sleep Project (HSP) [13] contains rich respiratory event information and can serve as a powerful supervision source for pre-training. Although PSG respiratory effort signals and radar-derived respiration waveforms differ in measurement physics, both modalities reflect the same underlying physiological process—thoracoabdominal motion dynamics during normal breathing and disordered events. This shared physiological substrate motivates cross-modality transfer learning from PSG to radar as a principled strategy to improve radar-domain performance. Compared with training on radar alone, PSG pre-training can provide a stronger inductive bias toward physiologically meaningful temporal patterns and improve data efficiency, especially when radar labels are scarce or noisy.

In this work, we investigate cross-modality transfer learning from PSG to FMCW radar for sleep apnea–hypopnea event detection under realistic data scarcity. Beyond proposing a U-Net-style encoder–decoder with a transformer bottleneck to capture long-range respiration dynamics [14,15,16], our key novelty lies in a transfer-and-evaluate framework that bridges the modality gap between contact-based respiratory effort belts and contactless radar respiration-motion waveforms. Specifically, we (i) pre-train a dense event segmentation model on large-scale PSG belt signals to learn generic event-related representations and then fine-tune it on synchronized FMCW radar recordings; (ii) formulate apnea/hypopnea detection as event-level dense segmentation with a unified probability-to-event conversion and consistent post-processing/matching rules, enabling fair and clinically interpretable comparisons across modalities; and (iii) provide a clinically grounded evaluation protocol that jointly reports event-level detection metrics (precision/recall/F1) and recording-level disease burden estimation (AHI and severity), together with ablations and robustness analyses that quantify when and why PSG-to-radar transfer is effective. Through comprehensive experiments, we aim to identify practical fine-tuning strategies for cross-domain adaptation and provide insights toward deployable, low-burden home sleep monitoring solutions.

The main contributions of this study are as follows:

1. Cross-modality transfer framework. We propose a PSG→FMCW radar transfer learning pipeline for apnea–hypopnea event detection under radar data scarcity, leveraging PSG pre-training and radar fine-tuning.
2. Event-level dense segmentation. We formulate detection as dense event segmentation with unified probability-to-event conversion and consistent post-processing/matching, achieving an event-level F1-score of 0.8167±0.0052 for radar.
3. Clinically grounded evaluation. We report clinically relevant metrics at both the event level (precision/recall/F1) and the recording level (AHI/severity) and provide ablations/robustness analyses to characterize transfer effectiveness.

## 2. Related Work

### 2.1. Radar SAHS Detection

Radar-based respiration sensing has long been explored as a non-contact approach for monitoring thoracoabdominal motion, enabled by advances in Doppler radar, ultra-wideband (UWB) radar, and, FMCW millimeter-wave systems [9,10]. A 2025 systematic review and network meta-analysis of radar-based obstructive sleep apnea (OSA) detection (20 studies, 1540 participants) reported that diagnostic performance varies markedly with frequency band, radar configuration (e.g., dual-radar setups), sensing distance, and the adoption of machine learning and underscored the substantial heterogeneity in study protocols and cohorts [17].

Across modalities, radar-based apnea detection typically follows a pipeline comprising: (i) range/phase processing to isolate subject-related returns; (ii) clutter suppression and mitigation of motion artifacts; (iii) extraction of a respiration surrogate (e.g., phase-demodulated displacement, micro-Doppler signatures, or amplitude/phase trajectories); and (iv) event inference. Foundational systems such as Vital-Radio demonstrated robust respiration extraction in realistic indoor settings using FMCW principles while highlighting practical challenges, including multipath propagation, interference, and body motion, that can degrade waveform fidelity and downstream detection [11].

Earlier studies often relied on hand-crafted, epoch-based features followed by conventional classifiers; while interpretable, performance can be brittle to changes in posture, sensing geometry, and environment, which is problematic for longitudinal home monitoring. A dedicated review on Doppler radar sleep monitoring summarizes common feature engineering and signal processing strategies and underscores the sensitivity of performance to motion artifacts and sensing geometry [18].

With the growing availability of synchronized PSG–radar recordings, deep learning has been increasingly adopted to reduce reliance on hand-crafted features and advance from coarse severity screening to event-level detection. A preliminary 60 GHz FMCW radar study employed convolutional recurrent architectures to detect apnea–hypopnea events, demonstrating the potential of sequence modeling while still being constrained by modest cohort sizes [19]. Event-centric formulations have also been explored: one study performed event-level identification from FMCW radar using a U-Net backbone with attention modules, reflecting a broader shift toward segmentation-based modeling that better matches clinically defined respiratory events than epoch-level classification [15]. Complementarily, large-scale respiratory-motion modeling has begun to inform radar research: a recent universal framework (ResSleepNet) was trained on large thoracoabdominal motion datasets for sleep staging and AHI estimation and then extended to radar-derived respiratory signals via transfer learning, explicitly positioning cross-modality pretraining as a way to mitigate radar label scarcity [20].

A recurring issue in radar OSA research is that evaluation targets vary: some works focus on AHI estimation or severity classification (screening), whereas others attempt event detection with temporal localization. Clinical validation studies illustrate the screening-oriented direction: a portable UWB radar device was validated for OSA screening against PSG, emphasizing feasibility outside sleep centers but also noting the lack of large-scale validations historically [21]. More recently, contact-free Doppler radar systems (e.g., SleepizOne+) have been evaluated in clinical cohorts with simultaneous PSG scoring, typically reporting diagnostic performance for AHI thresholds rather than event-wise segmentation accuracy [22]. In this context, ResSleepNet provides quantitative evidence that respiratory-motion pretraining can substantially improve radar-domain outcomes: pretraining on large-scale thoracoabdominal motion and fine-tuning on radar improved radar sleep-staging accuracy (61.6%→75.8%) and AHI estimation (ICC −0.13→0.87; MAE 22.97→8.80 events/h ) [20].

Taken together, the literature supports radar’s promise for unobtrusive sleep-disordered breathing assessment, but persistent gaps remain in dataset scale, protocol standardization, label consistency, and generalization across subjects and environments. These limitations motivate event-based modeling aligned with clinical definitions and the use of richer labeled source domains (e.g., PSG respiratory channels) to strengthen representation learning for radar.

### 2.2. Transfer Learning in Biosignal Processing

Transfer learning improves a target task by reusing knowledge learned from a related source task or domain. The classic taxonomy includes inductive transfer, transductive transfer (domain adaptation), and unsupervised transfer and highlights its particular value when target labels are scarce or costly, an archetypal setting in biosignal analytics [12]. In biomedical time series, limited labels are further compounded by inter-subject variability, device and preprocessing heterogeneity, and non-stationary physiology. Accordingly, transfer learning is widely used to improve generalization and reduce annotation demands. A 2014–2024 review summarizes common strategies, including (i) pre-training and fine-tuning, (ii) partial freezing with adaptation of task-specific heads, (iii) feature-level alignment/domain adaptation losses, and (iv) adversarial learning to mitigate domain shift [23].

Cross-modality transfer is generally more challenging than within-modality transfer because measurement physics and noise characteristics differ, but it can be effective when modalities reflect the same latent physiology and yield analogous temporal patterns (e.g., amplitude reductions and event boundary dynamics in apnea/hypopnea). This motivates PSG-to-radar transfer: PSG offers abundant clinically annotated respiratory channels, whereas radar datasets with synchronized labels remain limited. Public PSG repositories [13] provide scalable resources for representation learning even when downstream deployment uses a different sensing modality.

While transfer learning is well established in biosignal processing, and radar-based SAHS detection has progressed from feature engineering to deep learning, fewer studies have systematically explored PSG-to-radar cross-modality transfer for event-level respiratory event segmentation under clinically synchronized labels [20]. The broader radar SAHS evidence base indicates that performance is sensitive to radar configuration and cohort variability and that study heterogeneity remains high. This motivates a principled transfer pipeline: pre-training on large PSG respiratory channels to learn event-relevant temporal representations, followed by careful fine-tuning on radar respiration surrogates to address the target-domain shift and reduce overfitting.

## 3. Method

### 3.1. Overview of the Proposed Framework

**Figure 1:**
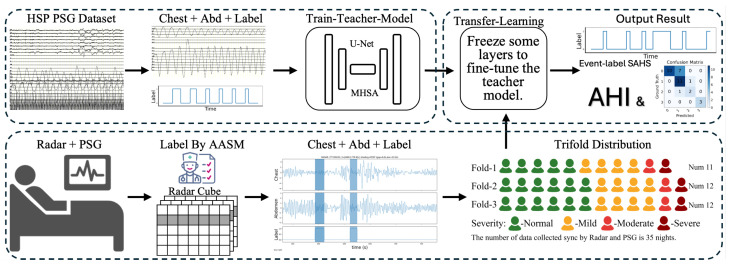
PSG-to-radar transfer-learning pipeline: train a teacher on HSP PSG and fine-tune on synchronized radar data to predict SAHS events and AHI (3-fold, 35 nights).

To enable accurate and scalable sleep apnea–hypopnea detection in home-like settings, we propose a two-stage cross-modality learning framework that transfers event-related knowledge from clinically established PSG respiratory signals to non-contact radar-derived respiration measurements. The pipeline is designed to address two practical constraints: PSG provides high-quality, clinically annotated events but is unsuitable for widespread longitudinal deployment, whereas radar supports unobtrusive monitoring but typically suffers from limited labeled data and substantial domain shift. An overview of the proposed two-stage cross-modality learning pipeline is illustrated in Figure 1. Accordingly, we leveraged large-scale PSG supervision for representation learning and then adapted the model to clinically synchronized radar data via fine-tuning. Specifically, the framework consists of the following:

1. PSG pre-training (source domain). We trained a sequence-to-sequence segmentation model on a large PSG cohort curated from the HSP, selecting 1526 overnight recordings with reliable respiratory annotations. The model mapped PSG respiratory effort signals (e.g., thoracic and abdominal channels) to dense per-sample probabilities of apnea/hypopnea-related events, learning general event morphology, temporal context, and intra-event dynamics expected to transfer across modalities.
2. Radar fine-tuning (target domain). We initialized the radar model with the PSG-pretrained weights and fine-tuned it on 35 overnight recordings collected at Beijing Tiantan Hospital with synchronized PSG-based annotations. Radar inputs were respiration motion waveforms extracted from mmWave measurements; fine-tuning adapted the representation to radar-specific variability while preserving event-relevant features learned from PSG.

Across both stages, the model output per-sample event probabilities rather than epoch-level labels, enabling event-centric inference and clinically meaningful endpoints. At test time, probabilities were thresholded and temporally post-processed (e.g., merging fragmented detections and filtering short segments) to produce event-level predictions. We report (i) event-level precision, recall, and F1-score to assess detection and localization accuracy and (ii) recording-level AHI estimation and severity classification to quantify screening utility. The subsequent Method Section details PSG curation/preprocessing and windowing, as well as radar waveform extraction, label alignment, and dataset splitting, mirroring the proposed two-stage pipeline.

### 3.2. PSG Data Preparation and Feature Extraction

PSG data were obtained from the HSP v2.0 hosted on the Brain Data Science Platform (BDSP). The HSP dataset contains large-scale, clinically acquired PSG studies (26,200 PSG studies from 19,492 patients) and provides standardized signal recordings and clinical annotations following AASM conventions. In HSP v2.0, PSG recordings include thoracic and abdominal respiratory effort channels, and signals are provided at (or resampled to) 200 Hz to enable synchronized multi-channel analyses [13].

Given the objective of learning apnea/hypopnea morphology for subsequent cross-modality transfer to radar, we curated an apnea/hypopnea-enriched subset from the full HSP repository. Specifically, we first selected nights with StudyType = “PSG Diagnostic” (diagnostic studies rather than titration or follow-up sessions) and then retained those with pre-sleep questionnaire information indicating evalForSleepApnea = 1, consistent with suspected SAHS evaluation. The HSP metadata provides these fields, enabling scalable cohort filtering.

Next, to ensure the AHI calculation corresponds to a physiologically meaningful sleep interval (and to reduce long wake segments that dilute event prevalence), we extracted the sleep segment from the first sleep onset to the final awakening. We then computed the AHI within the extracted interval and retained nights with AHI >4, which yielded 1526 nights for PSG pre-training [5]. The resulting AHI-based severity distribution of the curated HSP cohort (together with the target radar cohort) is summarized in Table 1, highlighting the underlying class composition and potential source–target imbalance.

These 1526 nights corresponded to 1252 unique subjects, with only a small fraction of subjects contributing multiple nights (typically 2–3 nights). This targeted curation improved the positive-sample density for supervised segmentation, thereby increasing the probability that each training window contained informative event morphology. In practice, this helped the model learn apnea/hypopnea-related temporal patterns more efficiently than training on a heavily imbalanced random sample of the full cohort.

We used the thoracic (chest) and abdominal respiratory effort belt channels as two-channel 1D inputs. HSP provides synchronized PSG signals with respiratory effort channels resampled to 200 Hz; in our pipeline, the channels were read from EDF and treated as time-aligned sequences (shared recording clock), allowing consistent label mapping to both inputs.

HSP annotations are provided in tabular form with entries defined by (epoch, time, duration, event), where time and duration are in seconds. We loaded the CSV, coerced numeric fields, removed missing/invalid rows (e.g., undefined time or non-positive duration), and retained respiratory-event descriptors. Because respiratory events may appear with two related prefixes (e.g., “Respiratory Event” and “RespEvent”) and include multiple subtypes, we implemented a robust parser that mapped event strings to integer codes via an explicit dictionary, as summarized in Table 2.

In this study, we formulated a binary segmentation task in which codes {1, 2, 3, 4} (obstructive apnea, central apnea, mixed apnea, and hypopnea) were treated as the positive class, whereas Normal (0) and OtherRespEvent (5) were treated as negative. OtherRespEvent captured respiratory-related annotations that were not scored as apnea/hypopnea under AASM rules and terminology [5], including Respiratory Effort-Related Arousal (RERA) and Partial Obstructive events, and was retained only for bookkeeping and optional secondary analyses.

To obtain per-sample supervision, each annotated interval $\left[t_{\mathrm{start}},t_{\mathrm{end}}\right]$ was rasterized into a label sequence aligned to the resampled respiratory signals. Several edge cases were handled to reduce label noise: (i) events that wrapped across midnight (end time < start time) were corrected by adding 24 h to the end time; (ii) overlapping events were resolved by a priority rule (apnea subtypes dominated hypopnea; otherwise, the longer event was retained); and (iii) adjacent events separated by ≤3 s were merged to avoid artificial fragmentation. These steps improved temporal continuity for sequence segmentation.

Although HSP respiratory effort belts are available at 200 Hz, apnea/hypopnea morphology is dominated by low-frequency respiration dynamics. We therefore downsampled both thoracic and abdominal belts to 10 Hz via integer-factor decimation (200 Hz→10 Hz), which also provided anti-aliasing filtering and reduced computational cost, while matching the radar respiration sampling rate used in the target domain. To mitigate inter-night variability from sensor placement and baseline drift, we applied night-wise robust normalization independently per channel: NaN/Inf values were replaced with zeros and signals were centered by the median, clipped to [median±4·IQR], and scaled by IQR (with a standard deviation fallback when the IQR was too small). The resulting integer label sequence was then binarized by marking codes in {1, 2, 3, 4} as positive and all others as negative.

Finally, each night was segmented into overlapping windows of 2048 samples with a stride of 300 samples (204.8 s windows with 30 s steps at 10 Hz), yielding $X\in R^{N\times 2048\times 2}$ and $y\in R^{N\times 2048\times 1}$. This windowing provided a long temporal context for pre-event baseline and post-event recovery while maintaining a practical stride consistent with conventional PSG epoching. The radar dataset and its preprocessing, including waveform extraction and PSG synchronization, are described next.

### 3.3. Radar Data Preparation and Feature Extraction

Radar respiration data were collected at Beijing Tiantan Hospital using a 60 GHz FMCW radar (Texas Instruments, Dallas, TX, USA), yielding 35 overnight recordings acquired from 35 independent subjects (one night per subject). Synchronized PSG was acquired in parallel for each subject-night. Specifically, the radar front-end was implemented with the Texas Instruments (Dallas, TX, USA) IWR6843ISK evaluation board together with a DCA1000 EVM data acquisition card; raw data were streamed in real time to a bedside computer and synchronized with PSG via timestamps. PSG technicians/clinicians provided time-aligned respiratory event annotations, which were then used as the ground truth for radar learning. This synchronized acquisition enabled training and evaluation under clinically consistent labeling, reducing ambiguities that often arise in radar-only studies. Key radar acquisition parameters are summarized in Table 3.

**Figure 2:**
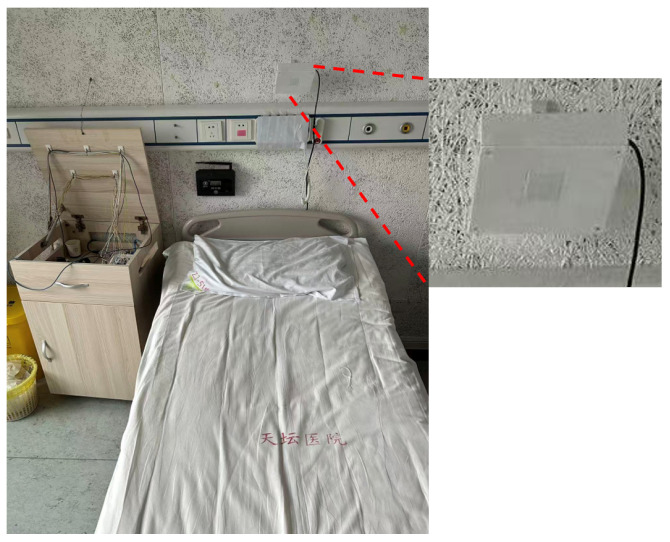
Photograph of the real-world data collection environment at Beijing Tiantan Hospital, showing the patient room setup and the installation position of the FMCW radar.

The real-world ward environment at Beijing Tiantan Hospital and the radar installation position during data acquisition are shown in Figure 2.

In the following, we describe the radar signal processing pipeline that transformed raw radar returns into two respiration-related waveforms (radar-chest and radar-abd) and then produced temporally aligned binary event labels for model fine-tuning. The overall pipeline is consistent with common FMCW-based respiration extraction practice and follows the processing logic implemented in our scripts.

For an FMCW radar transmitting a linear chirp, a standard complex-baseband formulation can be written as:

(1) $$s_{T}\left(t\right)=A_{T}\exp\left(j\left(2\pi f_{c}t+\pi St^{2}\right)\right),$$

where $f_{c}$ is the carrier frequency and $S=B/T_{c}$ denotes the chirp slope (bandwidth *B*, chirp duration $T_{c}$). The received signal from a dominant range bin experiences a round-trip delay τ(t)=2R(t)/c, and, after mixing/dechirping with the transmitted signal, the intermediate-frequency (IF) signal can be expressed (up to constants) as:

(2) $$s_{IF}\left(t\right)\propto \exp\left(j\left(2\pi S\tau t+\phi (t)\right)\right),$$

where the residual phase term ϕ(t) contains fine motion information. When chest wall motion induces a small displacement d(t) around a nominal range $R_{0}$, the phase modulation approximately satisfies:

(3) $$\Delta \phi \left(t\right)\approx \frac{4\pi }{\lambda }d\left(t\right),\;d\left(t\right)\approx \frac{\lambda }{4\pi }\mathrm{unwrap}\left(∠z(t)\right),$$

with wavelength $\lambda =c/f_{c}$ and z(t), the complex signal of the selected range bin (or a linear combination of bins). This phase-to-displacement conversion and phase unwrapping operation is explicitly used in our implementation.

After standard radar front-end processing (e.g., range FFT on I/Q samples), we obtained a range–time representation (or an equivalent “FFT cube”) in which each range bin provided a complex-valued slow-time sequence. Because respiration-induced micro-motion can be distributed across neighboring bins (due to multipath, posture changes, and torso extent), using multiple bins can improve robustness; prior FMCW apnea studies similarly emphasize that different range bins may contain complementary respiration information.

In our dataset generation, we retained 40 torso-related range bins for subsequent motion extraction. Concretely, each recording stores an array fft-cube whose second dimension equals 40 (bins), serving as the multi-bin input for thoracoabdominal waveform reconstruction. Given the complex slow-time signal for each bin, we first removed per-bin DC components and then computed the unwrapped phase to recover continuous displacement trajectories. In code, this corresponded to: (i) subtracting the mean complex value per bin, (ii) computing ∠z(t), (iii) applying np.unwrap along time, and (iv) converting phase to displacement using λ/(4π).

To isolate respiration dynamics, we applied a band-pass Butterworth filter targeting typical breathing frequencies. The implementation used a 4th-order Butterworth design and filtfilt to avoid phase distortion.

A key step was transforming the 40-bin displacement matrix into two respiration-related signals intended to approximate thoracic and abdominal components. Let $X\in R^{T\times B}$ be the matrix of filtered displacement signals (here, B=40).

We estimated two weight vectors $w_{c}$ and $w_{a}$, such that:

(4) $${\hat{y}}_{c}=Xw_{c},\;{\hat{y}}_{a}=Xw_{a},$$

where ${\hat{y}}_{c}$ and ${\hat{y}}_{a}$ correspond to radar-derived chest and abdomen motion signals.

We adopted a ridge regression solution with an additional orthogonality-promoting constraint between the two projections to encourage disentanglement of chest/abd motion contributions—specifically, ridge regression provides a stable estimator under multicollinearity:

(5) $$w=\arg\min_{w}\;{∥Xw-y∥}_{2}^{2}+\alpha {∥w∥}_{2}^{2}$$

and we further refined $\left(w_{c},w_{a}\right)$ to reduce correlation (approximate orthogonality) between the two weight vectors. This “orthogonal ridge” procedure was implemented in our script by iteratively solving ridge regression and projecting one weight vector onto the orthogonal complement of the other.

To improve temporal stability, weights were estimated within overlapping windows and then merged by overlap-add averaging (rather than fitting a single global mapping). The script used 120 s windows with a 30 s stride during waveform construction and aggregated overlapping predictions to form full-length radar-chest and radar-abd sequences.

After reconstruction, we applied an additional band-pass filter to suppress residual drift and high-frequency noise. The radar slow-time sampling rate in preprocessing was 50 Hz, and the respiration waveforms were downsampled to 10 Hz to match the PSG training interface and reduce computation; the 50 Hz→10 Hz conversion was implemented by decimation. To handle minor length mismatches across channels and labels (e.g., trimming or missing frames), we enforced per-night alignment by trimming radar-chest, radar-abd, and label sequences to the same minimum length.

We then applied the same robust per-night normalization used for PSG to radar-chest and radar-abd, including NaN/Inf handling, median–IQR scaling, and outlier clipping, which reduced inter-night amplitude variability and improved fine-tuning stability. Radar annotations followed the same categorical definition and codebook as PSG (Table 2); for the binary task, labels were binarized with {1, 2, 3, 4} as positive (apnea/hypopnea) and {0, 5} as negative.

Finally, radar sequences were segmented using the same sliding-window protocol as PSG—overlapping windows of 2048 samples with a stride of 300 samples (204.8 s windows with 30 s steps at 10 Hz)—yielding input tensors $X\in R^{N\times 2048\times 2}$ corresponding to the radar-chest and radar-abd channels and label tensors $y\in R^{N\times 2048\times 1}$ derived from synchronized PSG annotations. Overall, the preprocessing pipeline converted multi-bin FMCW slow-time complex signals into two normalized respiration-motion waveforms via phase-based displacement recovery, respiration-band filtering, and orthogonal ridge projection with overlap–add fusion, while standardizing sampling rate, channel count, labeling, and windowing to enable principled PSG-to-radar transfer learning.

### 3.4. Model Building and Architecture

#### 3.4.1. Task Formulation and PSG-to-Radar Transfer Design

We formulated apnea/hypopnea detection as a one-dimensional dense segmentation problem. For each sliding window, the input was a two-channel respiratory sequence $X\in R^{T\times 2}$ with T=2048, where the channels corresponded to thoracic- and abdominal-related respiratory motion (PSG effort belts in the source domain; radar-derived chest/abd motion in the target domain). The model output per-sample event probabilities $\hat{y}=\sigma \;\left(f_{\theta }\left(X\right)\right)\in {\left[0,1\right]}^{T\times 1}$, where $f_{\theta }\left(\cdot \right)$ denotes the network mapping and σ(·) is the sigmoid function. Dense segmentation was clinically aligned because respiratory events are defined by start/end boundaries and duration, which directly determine AHI and severity strata under standard scoring rules [5]. Compared with epoch-level classification (e.g., 30 s labels) [24,25], dense prediction better preserves within-event morphology and supports principled temporal post-processing (e.g., merging fragmented detections and enforcing minimum-duration constraints), improving event continuity and localization, consistent with event-level FMCW radar apnea identification frameworks [15].

To mitigate radar label scarcity and cross-modality domain shift, we trained the model in two stages: pre-training on 1526 PSG nights curated from the HSP to learn general respiratory-event representations, followed by fine-tuning on 35 synchronized PSG–radar nights collected at Beijing Tiantan Hospital. The architecture was designed for data-efficient transfer and robust temporal localization: we adopted a U-Net-style encoder–decoder to impose strong locality and boundary-recovery bias [14], enhance multi-scale temporal context via dilated convolutions and ASPP-style aggregation [26,27], and incorporate a transformer bottleneck to capture long-range dependencies [16]. This design is intentionally aligned with recent event-level radar apnea models that combine convolutional segmentation backbones with attention mechanisms [15].

#### 3.4.2. Overall Network Topology

We employed an optimized 1D U-Net-style encoder–decoder for dense respiratory event segmentation, tailored to capture both (i) sharp event boundaries (onset/offset) and (ii) longer-range breathing dynamics (baseline–suppression–recovery). Given an input window $X\in R^{T\times 2}$ (T=2048), the encoder comprised L=4 stages with skip connections and progressively increased channel capacity (base filters =32) [14]. The U-Net topology provided a strong inductive bias for localization: deep encoder features encoded semantic context while skip fusion restored high-resolution cues for boundary reconstruction in the decoder, which is crucial for event-level apnea/hypopnea segmentation.

To complement convolutional locality, we incorporated a transformer bottleneck with multi-head self-attention (MHSA) at the lowest temporal resolution ($T_{b}=T/2^{L}=128$), where attention is computationally efficient and can explicitly model long-range dependencies within a window [16]. This is particularly beneficial for SAHS event segmentation because hypopnea/apnea patterns are often defined relative to preceding baseline breathing and subsequent recovery, and radar inputs may contain motion-induced artifacts that require global context to disambiguate. In our implementation, the bottleneck embeddings were augmented by sinusoidal positional encoding and processed by 3 prenorm transformer blocks (4 heads), improving temporal coherence under inter-night variability and cross-modality shift.

Optimization techniques enabled by our implementation. (1) Residual and dilated convolutions. We adopted residual Conv1D blocks in the encoder together with modest dilation (e.g., dilation rates (1,2)) to stabilize optimization and enlarge the effective receptive field without sacrificing temporal resolution. This design improves gradient flow during training and helps capture event morphology spanning tens of seconds, which is important for apnea/hypopnea segmentation [26,28]. (2) Learnable convolutional downsampling. Instead of fixed pooling, we performed downsampling using stride-2 convolutions, which provides a learnable anti-aliasing mechanism and better preserves waveform morphology under downsampling. This can reduce sensitivity to setup-dependent distortions and improve robustness when adapting from PSG to radar [29]. (3) ASPP-style multi-scale context aggregation. We incorporated an ASPP-lite module at the bottleneck with parallel dilated branches (e.g., (1,2,4,8)) to aggregate multi-scale temporal context prior to attention. This is well matched to the substantial variability in event duration and waveform signatures across apnea and hypopnea, enabling the model to represent both short irregularities and sustained suppressions within a unified segmentation framework [27].

**Figure 3:**
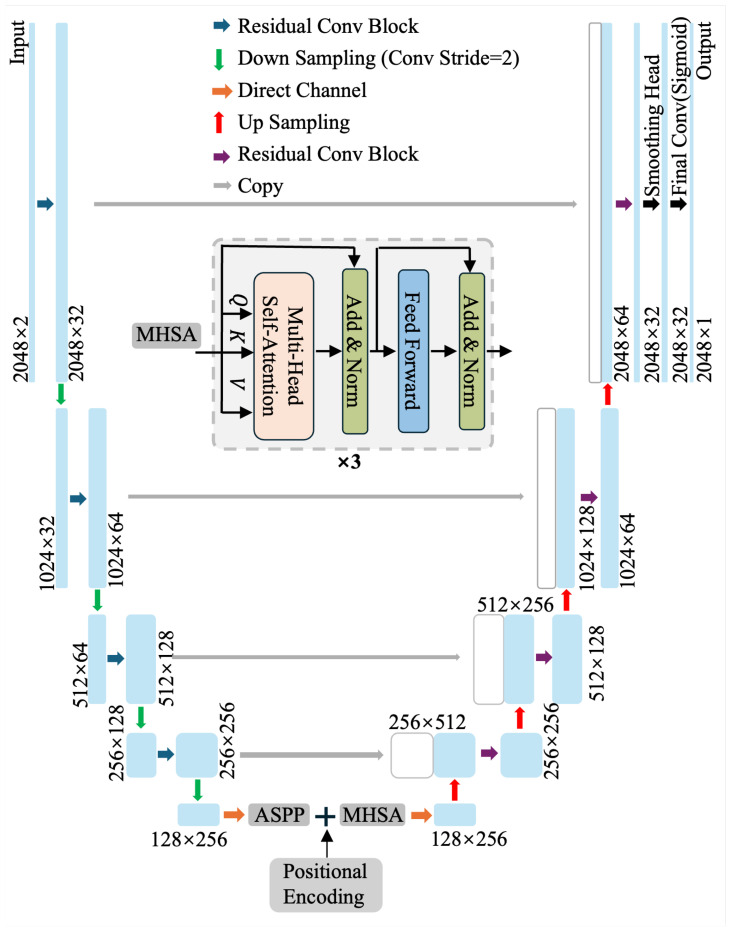
Proposed 1D U-Net–MHSA architecture with residual encoder–decoder, skip connections, ASPP+MHSA bottleneck, and a smoothing head with sigmoid output.

Figure 3 provides an overview of the architecture (Encoder–ASPP–Transformer Bottleneck–Decoder).

Unless otherwise stated, we used the following configuration in Table 4.

### 3.5. Loss and Optimization: Training Details

Loss function. We used a combination of weighted binary cross-entropy (BCE) and Dice loss to address severe class imbalance:

(6) $$L=\alpha \cdot L_{\mathrm{Dice}}+\left(1-\alpha \right)\cdot L_{\mathrm{wBCE}}$$

where the Dice loss was:

(7) $$L_{\mathrm{Dice}}=1-\frac{2\sum (y\hat{y})+\epsilon }{\sum y+\sum \hat{y}+\epsilon }$$

and the weighted BCE was:

(8) $$L_{\mathrm{wBCE}}=-E\left[w_{\mathrm{pos}}y\log\left(\hat{y}\right)+w_{\mathrm{neg}}\left(1-y\right)\log\left(1-\hat{y}\right)\right].$$

We set α=0.8, $w_{\mathrm{pos}}=2.0$, and $w_{\mathrm{neg}}=1.0$. Optimizer and hyperparameters. For PSG pre-training, we used Adam with learning rate $1\times 10^{-4}$ and gradient clipping (clipnorm = 1.0), batch size 96, and up to 80 epochs.

Callbacks and model selection. Because standard sample-level metrics may not reflect event detection quality, we monitored event-level F1 on validation nights using a sliding-window fusion evaluation callback and selected the best model checkpoint using val-event-f1. Early stopping and learning-rate scheduling were applied to stabilize training.

### 3.6. Transfer Learning Radar Fine-Tuning

In the target domain, we adapted the PSG-pretrained event segmentation network to non-contact respiration waveforms derived from a 60 GHz FMCW radar. Due to the limited scale of clinically labeled radar data (35 overnight recordings collected at Beijing Tiantan Hospital with PSG-synchronized annotations), we adopted a transfer learning strategy that preserved modality-invariant respiratory dynamics learned from large-scale PSG while allowing higher-level representations to adjust to radar-specific noise, motion artifacts, and domain shift. Similar to prior radar-based event-level apnea identification pipelines, we treated radar apnea detection as a sample-wise segmentation problem followed by event extraction via temporal post-processing.

#### 3.6.1. Teacher Initialization and Configuration Consistency

Let $f_{\theta }\left(\cdot \right)$ denote the segmentation model parameterized by θ. In the source domain, we obtained a PSG teacher checkpoint $\theta_{\mathrm{PSG}}^{★}$ by minimizing the event segmentation loss described in Section 3.5. In the target domain, the radar model was initialized by weight transfer,

(9) $$\theta_{0}\leftarrow \theta_{\mathrm{PSG}}^{★},$$

and then optimized on radar windows. To ensure architectural equivalence between the teacher and the student, we loaded the teacher-side model configuration (e.g., residual blocks, convolutional downsampling, ASPP-lite, transformer bottleneck) from a serialized configuration file and reconstructed the identical network before loading weights. This practice avoids silent mismatches that may occur when only partial hyperparameters are reused and is recommended for reproducible transfer learning in deep networks.

#### 3.6.2. Layer Freezing and Normalization Stabilization

Directly fine-tuning all layers can overfit rapidly on small radar cohorts. We therefore employed partial layer freezing: a fixed proportion of early layers were set as non-trainable, while deeper layers (decoder, bottleneck, and head) remained trainable to adapt to radar characteristics. Denoting the ordered layer set as $\left\{\ell_{1},\ldots ,\ell_{L}\right\}$, we froze the first ρL layers (ρ∈(0,1)),

(10) $$\ell_{i}\;\mathrm{frozen},\;i\leq \left\lfloor \rho L\right\rfloor ,$$

and fine-tuned the remainder. Here, ρ is treated as a tunable training hyperparameter that controls the proportion of layers frozen during fine-tuning; based on an ablation over candidate values, we set ρ=0.6 as the default since it yielded the best overall validation performance in our experiments.

In addition, we froze all batch normalization (BN) layers [30] during radar fine-tuning. BN relies on mini-batch statistics and maintains running estimates of mean/variance; when the target dataset is small and the effective batch statistics differ from the source domain, updating BN can introduce instability and degrade transfer performance. Freezing BN is a commonly used stabilization heuristic in low-data fine-tuning settings and has been shown to improve robustness under domain shift.

#### 3.6.3. Fine-Tuning Objective and Optimization Details

Radar fine-tuning started from the PSG-pretrained teacher checkpoint and adapted the model to FMCW radar respiration-motion waveforms under limited radar labels. We used the same imbalance-aware segmentation loss as in PSG pre-training (Section 3.5):

(11) $$L_{\mathrm{radar}}\left(\theta \right)=\alpha \;L_{\mathrm{Dice}}+\left(1-\alpha \right)\;L_{\mathrm{wBCE}},$$

where α balances overlap-sensitive Dice loss and weighted binary cross-entropy for probabilistic calibration. Dice-style losses provided stronger gradients when apnea/hypopnea events occupied only a small fraction of the timeline [31], while the weighted BCE term helped stabilize early optimization and avoid degenerate all-negative predictions.

(1) Layer freezing and normalization

To reduce overfitting and catastrophic forgetting under the small radar cohort, we fine-tuned only a subset of layers by freezing a fraction ρ of earlier layers. In our default setting, we set ρ=0.6 and kept BatchNorm layers frozen during fine-tuning, as updating BN statistics with small radar batches can be unstable under distribution shift.

(2) Optimization, scheduling, and model selection

We optimized $L_{\mathrm{radar}}$ using Adam with a smaller learning rate than that for PSG pre-training:

(12) $$\theta \leftarrow \theta -\eta \;\mathrm{Adam}\left(\nabla_{\theta }L_{\mathrm{radar}}\right),\;\eta =5\times 10^{-5}.$$

Radar fine-tuning was conducted with batch size 96 for up to 50 epochs. We monitored the validation loss on the held-out fold and applied early stopping; the checkpoint with the best validation loss was used for subsequent evaluation and post-processing. To mitigate variance under limited data, fine-tuning and evaluation were performed within the K=3 severity-stratified cross-validation protocol (Section 3.6.4).

(3) Training windows and supervision.

Radar inputs were segmented into overlapping fixed-length windows (same window length as PSG pre-training) with a fixed step size, and the model was supervised with clinically synchronized PSG event labels mapped to the radar timeline. This ensured that optimization was performed at the dense (per-timestep) segmentation level while evaluation was reported at clinically meaningful event and recording levels (Section 3.7).

#### 3.6.4. Three-Fold Cross-Validation with AHI-Stratified Radar Folds and Cohort Severity Summary

To reduce variance caused by the small target radar cohort, we employed K=3-fold cross-validation on the radar nights. Each overnight recording was assigned an AHI-based severity label (normal, mild, moderate, severe) according to standard clinical thresholds [5]. Table 1 summarizes the AHI-based severity distributions for both the source PSG (HSP) cohort and the target radar cohort, which improves transparency of cohort imbalance and highlights potential source–target distribution mismatch relevant to cross-modality transfer.

In particular, the radar cohort contained only 35 nights and was imbalanced (normal: 17, mild: 12, moderate: 3, severe: 3), so we adopted K=3 as a practical compromise between training-set size per fold and the ability to perform severity-stratified splitting. Specifically, we constructed radar folds via severity-stratified allocation and approximately distributed each class across three folds, which yielded more balanced held-out sets than a single random split and provided a more reliable estimate of generalization under limited sample size. In fold *k*, we trained on the union of the other folds and evaluated on the held-out fold. As a result, the per-fold radar severity composition was approximately balanced, with class counts (code 0/1/2/3) of 5/4/1/1 for one fold and 6/4/1/1 for the other two folds.

#### 3.6.5. Model Selection and Training Callbacks Based on Event-Level F1

For apnea/hypopnea detection, sample-wise accuracy is often dominated by non-event segments and may not reflect clinical utility. We therefore selected checkpoints using event-level F1 computed on held-out nights by sliding inference and window-fusion, consistent with event-centric evaluation protocols. Concretely, after each epoch, we ran inference on validation nights, merged overlapping window predictions, applied temporal post-processing (Section 3.7), and computed event-level precision/recall/F1. The checkpoint with maximal validation event-F1 was retained:

(13) $$\theta_{\mathrm{radar}}^{★}=\arg\max_{\theta \in \{\theta_{e}\}}F1_{\mathrm{event}}\left(\theta \right),$$

where $\theta_{e}$ is the model state at epoch *e*. We further employed early stopping on event-F1 (patience =3, with best weights restored) and reduced the learning rate on plateaus of validation loss (factor =0.5, patience =2, minimum learning rate $=10^{-6}$) to stabilize training.

### 3.7. Post-Processing and Metrics

We evaluated the proposed PSG-to-radar transfer framework at three complementary levels: (i) sample-level discrimination (point-wise segmentation quality), (ii) event-level detection (clinical respiratory-event identification), and (iii) recording-level severity estimation via the AHI. Following recent event-centric radar apnea studies, we converted the model’s per-sample probability output into temporally consistent event intervals using a lightweight post-processing module and then computed event/recording metrics under a standardized matching protocol.

#### 3.7.1. Sliding-Window Fusion for Full-Night Inference

Let a full-night two-channel respiration recording be denoted as $X\in R^{L\times 2}$ with length *L* samples and sampling frequency $f_{s}$ (Hz). During inference, we applied a sliding window of length *T* (samples) and stride *S* (samples), producing *K* overlapping windows. For the *k*-th window starting at index $s_{k}$, the network output a probability sequence ${\hat{p}}^{\left(k\right)}\in {\left[0,1\right]}^{T}.$ To obtain a full-length probability sequence $\hat{p}\in {\left[0,1\right]}^{L}$, we fused overlapping predictions via unbiased averaging:

(14) $$\hat{p}\left[t\right]=\frac{\sum_{k=1}^{K}1\left(s_{k}\leq t<s_{k}+T\right)\;{\hat{p}}^{\left(k\right)}\left[t-s_{k}\right]}{\sum_{k=1}^{K}1\left(s_{k}\leq t<s_{k}+T\right)}\;,\;t=0,\ldots ,L-1,$$

where 1(·) is the indicator function. This fusion reduced boundary artifacts caused by windowing and stabilized probability trajectories over long recordings.

#### 3.7.2. Probability-to-Event Conversion and Temporal Regularization

Given the fused probability sequence $\hat{p}$, we first applied thresholding with τ∈(0,1):

(15) $$\tilde{y}\left[t\right]=1\left(\hat{p}\left[t\right]\geq \tau \right).$$

Contiguous positive samples formed preliminary event candidates $\tilde{E}={\left\{\left[a_{i},b_{i}\right)\right\}}_{i=1}^{N},$ where $a_{i}$ and $b_{i}$ denote event start and end indices.

Because radar/PSG respiratory-event segmentation may exhibit short spurious gaps and fragmented detections (e.g., due to noise, posture changes, or window-boundary effects), we applied two temporal regularizers:

(1) Event merging

For two consecutive events $\left[a_{i},b_{i}\right)$ and $\left[a_{i+1},b_{i+1}\right)$, we defined the inter-event gap $g_{i}=a_{i+1}-b_{i}.$ If the gap duration satisfied:

(16) $$\frac{g_{i}}{f_{s}}\leq g_{\min}\;,$$

we merged them into a single event $\left[a_{i},b_{i+1}\right)$. In this work, we set $g_{\min}=6\;s$ based on repeated empirical evaluation on the validation folds: smaller values tended to keep fragmented predictions as separate events (inflating false positives and increasing boundary jitter), whereas larger values increased the risk of over-merging truly distinct events. We found 6s to provide a robust trade-off that suppressed short “prediction jitter” gaps while preserving clinically distinct events.

(2) Minimum duration filtering.

After merging, any event with a duration shorter than $d_{\min}$ seconds was removed:

(17) $$\frac{b_{i}-a_{i}}{f_{s}}<d_{\min}\;\Rightarrow \;\left[a_{i},b_{i}\right)\;\mathrm{discarded}.$$

We adopted $d_{\min}=10\;s$ to align with the AASM respiratory-event scoring convention that apnea/hypopnea events must last at least 10 s [5], ensuring that the post-processing was clinically consistent and that the resulting event definitions matched the reference annotations.

#### 3.7.3. Event-Level Matching and Metrics

Let the post-processed predicted events be $\hat{E}={\left\{\left[{\hat{a}}_{m},{\hat{b}}_{m}\right)\right\}}_{m=1}^{\hat{N}}$ and the ground-truth events be $E={\left\{\left[a_{n},b_{n}\right)\right\}}_{n=1}^{N}.$ We matched predicted and true events using interval intersection-over-union (IoU), commonly used in event segmentation:

(18) $$\mathrm{IoU}\left(\left[\hat{a},\hat{b}\right),\left[a,b\right)\right)=\frac{\left|\left[\hat{a},\hat{b}\right)\cap \left[a,b\right)\right|}{\left|\left[\hat{a},\hat{b}\right)\cup \left[a,b\right)\right|}.$$

A predicted event was counted as a true positive (TP) if it could be uniquely matched to a ground-truth event with IoU>θ (we used θ=0.1 unless otherwise stated), using one-to-one matching to prevent double counting. Unmatched predictions and labels were false positives (FPs) and false negatives (FNs), respectively. Event-level precision, recall, and F1 were:

(19) $$\mathrm{Precision}=\frac{\mathrm{TP}}{\mathrm{TP}+\mathrm{FP}},\;\mathrm{Recall}=\frac{\mathrm{TP}}{\mathrm{TP}+\mathrm{FN}},\;F1=\frac{2\;\mathrm{Precision}\cdot \mathrm{Recall}}{\mathrm{Precision}+\mathrm{Recall}}.$$

#### 3.7.4. Recording-Level AHI Estimation and Severity Grading

For a recording with analyzed sleep duration (in samples) *L* and sampling frequency $f_{s}$, the predicted AHI was estimated from the number of predicted events $\hat{N}$:

(20) $$\hat{\mathrm{AHI}}=\frac{\hat{N}}{L/(3600\;f_{s})}\;\left(\mathrm{events}/\mathrm{hour}\right).$$

Across recordings, we report AHI mean absolute error (MAE) and root mean squared error (RMSE):

(21) $${\mathrm{MAE}}_{\mathrm{AHI}}=\frac{1}{M}\sum_{i=1}^{M}\left|{\hat{\mathrm{AHI}}}_{i}-{\mathrm{AHI}}_{i}\right|,\;{\mathrm{RMSE}}_{\mathrm{AHI}}=\sqrt{\frac{1}{M}\sum_{i=1}^{M}{\left({\hat{\mathrm{AHI}}}_{i}-{\mathrm{AHI}}_{i}\right)}^{2}},$$

and correlation between estimated and reference AHI (Pearson and Spearman).

For severity grading, we used standard AASM cutoffs [5]:

(22) Normal:AHI<5,Mild:5≤AHI<15,Moderate:15≤AHI<30,Severe:AHI≥30.

We report 4-class accuracy and Cohen’s κ to account for chance agreement:

(23) $$\kappa =\frac{p_{o}-p_{e}}{1-p_{e}},$$

where $p_{o}$ is the observed agreement and $p_{e}$ is the expected agreement under independence.

## 4. Results

### 4.1. Overall Performance

We evaluated PSG→Radar transfer learning on the radar dataset (35 overnight recordings from 35 independent subjects, one night per subject) using 3-fold cross-validation with AHI-stratified folds (normal/mild/moderate/severe) to reduce variance under the limited cohort size and mitigate severity distribution shift across splits. In each fold, we trained on two folds and evaluated on the held-out fold; because each subject contributed only one night, this protocol corresponded to a subject-independent evaluation. Fold-specific train/val file lists were exported to ensure reproducibility. All windows extracted from the same night were kept within the same fold to prevent any within-night data leakage. We report the mean ± standard deviation across folds for both event-level and recording-level metrics. Table 5 compares the proposed teacher-initialized fine-tuning (initialized from the PSG teacher checkpoint) with radar-only training from scratch under the same architecture and optimization settings. According to Table 6, ρ=0.6 and ρ=0.7 performed comparably well among ρ∈0.5,0.6,0.7,0.8; because our training/model selection criterion was event-level F1, we adopted ρ=0.6 as the best model in the following overall comparison. Overall, teacher initialization yielded substantially improved event-level detection (P/R/F1) and lower recording-level AHI errors (MAE/RMSE), indicating that PSG pretraining provided transferable respiratory-event representations despite the modality shift to radar.

**Figure 4:**
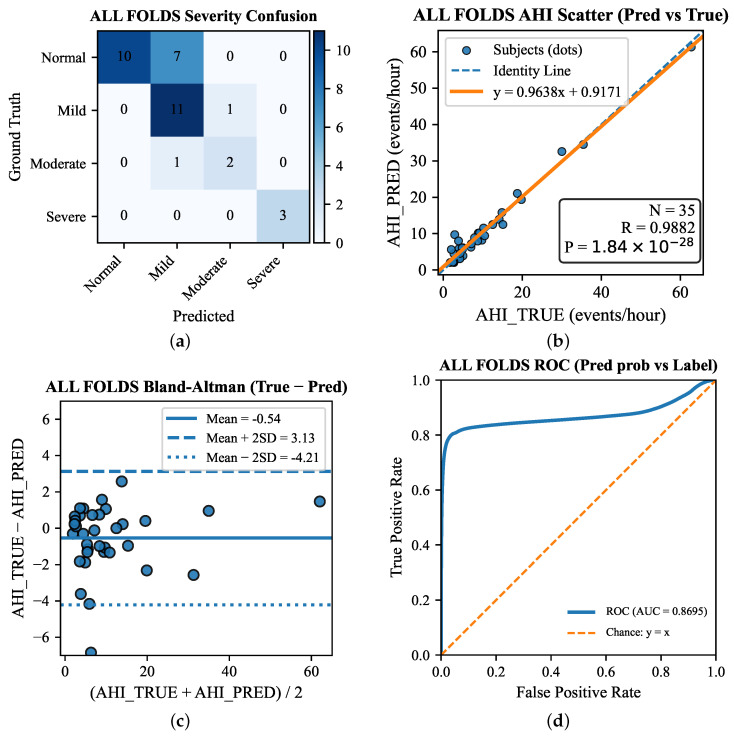
Overall-fold visualization of clinical agreement. (**a**) Severity confusion matrix. (**b**) AHI scatter plot (Pred vs. True). (**c**) Bland–Altman plot (True − Pred). (**d**) ROC curve (Pred prob vs. Label).

To complement scalar metrics, Figure 4 visualizes clinical agreement, discrimination, and error patterns across all folds. Specifically, Figure 4a reports the severity confusion matrix to characterize misclassification structure; Figure 4b,c evaluates recording-level AHI agreement using the predicted-versus-reference scatter plot and Bland–Altman analysis, respectively; and Figure 4d presents the ROC curve to summarize event-level discrimination based on predicted probabilities versus labels.

### 4.2. Ablation Study on Transfer Learning

We conducted an ablation study to quantify the contribution of key transfer learning choices during radar fine-tuning, including (i) teacher initialization vs. training from scratch, (ii) sensitivity to the layer-freezing ratio ρ, and (iii) freezing normalization layers. For teacher initialization, we compared fine-tuning initialized from the PSG teacher checkpoint against scratch training under identical architecture and optimization settings; in the teacher-initialized setting, we loaded the teacher weights and applied partial layer freezing, whereas scratch training disabled freezing (ρ=0) to avoid unnecessary constraints. Overall, teacher initialization improved convergence stability and generalization, supporting the hypothesis that PSG pretraining captures transferable respiratory dynamics despite cross-modality shift. To examine the amount of freezing needed, we swept ρ∈{0.5,0.6,0.7,0.8} and observed a non-monotonic (“single-hump”) trend, indicating that an intermediate freezing regime best balanced preserving generic event representations with adapting to radar-specific characteristics; excessive freezing tended to under-adapt, whereas insufficient freezing could overfit the small radar cohort. Finally, we ablated freezing normalization layers (BatchNorm) during fine-tuning: keeping BN frozen yielded better performance than updating BN statistics, consistent with the instability of BN estimates under small radar batches and distribution shift, which could degrade calibration and downstream event post-processing. Table 6 summarizes the results.

#### Quantifying PSG–Radar Feature-Space Alignment Before and After Fine-Tuning

**Figure 5:**
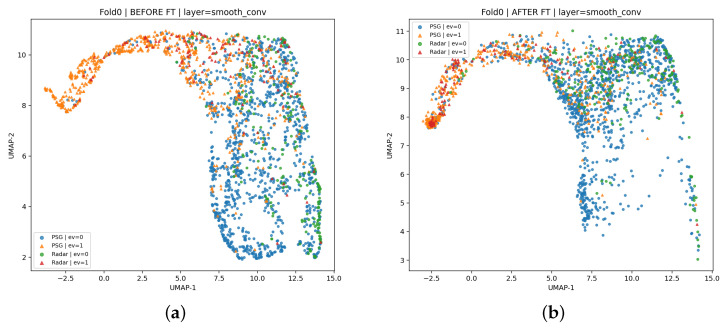
UMAP visualization of PSG and radar features at layer smooth_conv. (**a**) Before fine-tuning. (**b**) After fine-tuning. The UMAP embedding was fitted on pre-fine-tuning features, and post-fine-tuning features were projected using the same scaler and UMAP reducer for direct comparison.

To address the cross-modality domain shift between PSG and FMCW radar, we further provide both qualitative visualization and quantitative measurements of the feature-space discrepancy before and after fine-tuning. Specifically, we extracted intermediate representations from the fine-tuned radar model (layer: smooth_conv) and visualized them with UMAP (Figure 5). To ensure a fair before/after comparison, we fit the feature standardization and UMAP embedding on the pre-trained (before fine-tuning) features and projected the post-fine-tuning features using the same scaler and UMAP reducer.

In addition to visualization, we quantified the PSG–radar modality gap using multiple complementary metrics computed on the extracted features: (i) a linear domain-probe classifier AUC (lower is better; 0.5 indicates maximal domain confusion), (ii) distribution discrepancy via ${\mathrm{MMD}}^{2}$ (lower is better), (iii) second-order statistic difference via CORAL (lower is better), and (iv) local neighborhood mixing ratio based on *k*-NN (higher is better). As summarized in Table 7, all metrics consistently indicated reduced feature-space discrepancy after fine-tuning.

### 4.3. Ablation Study on Model Components

To quantify the contribution of long-range temporal modeling, we explicitly included a CNN-only baseline, i.e., a pure 1D U-Net (Base U-Net), and compared it against the proposed U-Net+MHSA with a transformer bottleneck. The Base U-Net served as a standard and strong segmentation baseline for dense sample-wise labeling, relying solely on convolutional receptive fields to capture event morphology. In contrast, the MHSA bottleneck explicitly modeled non-local temporal dependencies that were relevant to the baseline–suppression–recovery dynamics across an event and its surrounding context. Empirically, removing MHSA led to increased event fragmentation and boundary jitter and reduced robustness to transient motion artifacts, resulting in degraded event-level performance. The quantitative comparison between Base U-Net and this study is reported in Table 8.

### 4.4. Ablation on Targeted Training Techniques

We further investigated the contribution of three targeted design techniques used in our implementation, namely, (i) residual learning with local dilated temporal modeling, (ii) learnable convolutional downsampling for morphology-preserving multi-scale representation, and (iii) ASPP-style multi-scale context aggregation. To assess their joint effect, we compared the full configuration against a simplified variant in which these techniques were removed simultaneously. Overall, disabling the three techniques led to a consistent degradation in event-level detection performance and/or AHI estimation stability, indicating that these complementary mechanisms collectively improve robustness to multi-scale respiratory morphology, boundary uncertainty, and radar-domain noise. Table 9 summarizes the results.

## 5. Conclusions and Discussion

### 5.1. Summary and Conclusions

Non-contact FMCW radar-based apnea/hypopnea event detection remains challenging due to cross-modality domain shift relative to PSG, sensitivity to environmental factors, and limited clinically synchronized radar labels. To address these constraints, we presented a cross-modality transfer-and-evaluate framework that leveraged large-scale PSG respiratory effort recordings to pre-train dense event representations and adapt them to radar respiration-motion waveforms via fine-tuning.

Concretely, a 1D encoder–decoder segmentation model with long-range temporal modeling was pre-trained on 1526 PSG nights from the HSP dataset and fine-tuned on 35 synchronized PSG–radar nights collected at Beijing Tiantan Hospital. Under the radar evaluation protocol, PSG→Radar transfer consistently improved event-level detection and AHI estimation compared with radar-only training.

From a clinical perspective, the achieved event-level F1 (about 0.82) should be interpreted in light of the known subjectivity of respiratory-event scoring: even among trained scorers, hypopnea and event-type labeling exhibited only moderate agreement, which sets a practical ceiling on model–PSG concordance. Moreover, our AHI errors (MAE/RMSE; Table 5) were small relative to standard severity cutoffs (5/15/30 events·h^−1^), suggesting that the main practical risk of misclassification was concentrated near category boundaries. Taken together, these results indicate that PSG-initialized radar fine-tuning can provide clinically meaningful screening and longitudinal monitoring signals, while PSG remains the reference for definitive diagnosis.

### 5.2. Limitations

Despite the promising PSG→radar transfer results for event-level apnea/hypopnea segmentation, several limitations remain. First, the radar cohort was small (35 nights) and collected at a single center with one bedroom/sensor configuration, which may limit generalizability to home settings. Variations in room layout/clutter, radar–bed distance/angle, bedding-induced multipath, and posture patterns/turning can change radar micro-motion signatures and respiration SNR, leading to feature shift and performance variation. Second, our radar respiration extraction and synchronization benefit from controlled acquisition and clinically synchronized PSG, and may require more robust self-calibrating preprocessing and signal-quality control for fully reference-free deployment. Third, PSG hypopneas may be scored based on desaturation/arousal criteria that are weakly reflected in motion signals, causing label ambiguity and modality mismatch [5]. Future work will validate multi-site cohorts and improve robustness via calibration/normalization, augmentation, and domain adaptation.

### 5.3. Future Work

Future work will focus on improving generalization, deployment readiness, and clinical interpretability of the proposed framework. We plan to expand synchronized PSG–radar cohorts across multiple centers and home-like environments to better quantify robustness to sensing geometry, posture variability, and demographic heterogeneity and report subgroup-specific uncertainty. To enable practical reference-free operation, we will develop self-calibrating radar preprocessing, including adaptive bin selection, improved clutter/multipath suppression, and learned separation of respiration from motion artifacts. On the learning side, we will explore self-supervised or contrastive pretraining on large unlabeled radar recordings and investigate hybrid schemes that combine PSG supervision with radar self-supervision to further reduce label dependence and improve domain robustness. Methodologically, we will extend the current binary segmentation pipeline toward subtype-aware and multi-task formulations and examine explicit domain adaptation, test-time adaptation, and lightweight personalization to mitigate strong inter-subject and inter-environment shift. Finally, we will pursue prospective clinical studies to evaluate screening utility and longitudinal tracking performance in real home monitoring workflows, together with usability, privacy, and regulatory considerations for translation. 

## Figures and Tables

**Table 1:** Cohort size and AHI-based severity distribution for the source PSG (HSP) cohort and the target radar cohort.

Severity	Code	Radar	Radar (%)	HSP	HSP (%)
Normal	0	17	48.6	159	10.4
Mild	1	12	34.3	937	61.4
Moderate	2	3	8.6	332	21.8
Severe	3	3	8.6	98	6.4
Total	–	35	100.0	1526	100.0

**Table 2:** Respiratory event label.

Respiratory Event	Label
Normal	0
ObstructiveApnea	1
CentralApnea	2
MixedApnea	3
Hypopnea	4
OtherRespEvent	5

**Table 3:** Radar parameters of the 60 GHz FMCW system.

Parameter	Value	Parameter	Value
Start Frequency	60 GHz	Range Resolution	4.51 cm
Chirp Slope	65 MHz/μs	Chirp Rate	50 chirps/s
ADC Sample Rate	5 MHz	Transmission Power	10 dBm
Number of ADC Samples	256	Bandwidth	3.3 GHz

**Table 4:** Model configuration parameters.

Module	Parameter	Value
Global Architecture	Input shape/Output shape	2048×2/2048×1
Base Network	Base filters/Encoder depth/Dropout	32/4/0.2
Feature Extractor	Block type	Residual Conv Block
	Kernel size	3
	Dilation rates	(1,2)
	Normalization	BatchNorm
	Activation	Swish
	Downsampling	Strided convolution
	Upsampling	Nearest + Conv
Bottleneck	ASPP dilations	(1,2,4,8)
	Transformer blocks/Heads	3/4
	Embed dim/FFN multiplier	256/4
	Positional encoding	Sinusoidal
Output Head	Smoothing	Large-kernel conv (k=31)
	Final activation	Sigmoid

**Table 5:** Overall performance comparison on the radar dataset (mean ± std across 3 folds).

Model	Pretraining	Fine-Tune	Precision	Recall	F1	AHI MAE	AHI RMSE
Proposed (PSG→Radar)	PSG	Yes	0.8137±0.0332	0.8369±0.0470	0.8167±0.0052	1.3493±0.2165	1.8599±0.4112
Radar-only (scratch)	None	Yes	0.6807±0.0364	0.5773±0.0583	0.6059±0.0245	2.2368±0.5203	2.9398±0.4882

**Table 6:** Ablation on transfer learning strategy (mean ± std across 3 folds).

Ablation Factor	Setting	Init	ρ	BN Freeze	Precision	Recall	F1	AHI MAE	AHI RMSE
Teacher init vs. scratch	Fine-tune (teacher init)	Teacher (PSG)	0.6	Yes	0.8137±0.0332	0.8369±0.0470	0.8167±0.0052	1.3493±0.2165	1.8599±0.4112
	Train from scratch	Scratch	0.0	Yes	0.7597±0.0468	0.7975±0.0467	0.7675±0.0136	1.4356±0.4558	1.9645±0.3625
Freezing ratio sweep	Fine-tune (ρ=0.5)	Teacher (PSG)	0.5	Yes	0.7815±0.0115	0.8163±0.0108	0.7915±0.0089	1.1764±0.1470	1.8113±0.2236
	Fine-tune (ρ=0.6)	Teacher (PSG)	0.6	Yes	0.8137±0.0332	0.8369±0.0470	0.8167±0.0052	1.3493±0.2165	1.8599±0.4112
	Fine-tune (ρ=0.7)	Teacher (PSG)	0.7	Yes	0.8161±0.0302	0.8330±0.0337	0.8166±0.0043	1.1960±0.2090	1.7550±0.2458
	Fine-tune (ρ=0.8)	Teacher (PSG)	0.8	Yes	0.7973±0.0217	0.8393±0.0323	0.8092±0.0057	1.3277±0.1639	1.9412±0.1657
Normalization layers	Fine-tune (BN frozen)	Teacher (PSG)	0.6	Yes	0.8137±0.0332	0.8369±0.0470	0.8167±0.0052	1.3493±0.2165	1.8599±0.4112
	Fine-tune (BN trainable)	Teacher (PSG)	0.6	No	0.8129±0.0284	0.8274±0.0488	0.8122±0.0070	1.1733±0.1656	1.7282±0.2330

**Table 7:** Quantification of PSG–radar feature-space discrepancy before vs. after fine-tuning (layer: smooth_conv). Domain-probe AUC is reported as mean ± std from 5-fold cross-validation. Lower is better for AUC/${\mathrm{MMD}}^{2}$/CORAL; higher is better for *k*NN mixing.

Metric	Before FT	After FT
Domain-probe AUC (↓)	0.793±0.029	0.767±0.036
${\mathrm{MMD}}^{2}$ (↓)	0.0528	0.0143
CORAL (↓)	14.26	13.90
*k*NN mixing (↑)	0.203	0.232

**Table 8:** Ablation on architecture components (radar evaluation; mean ± std across folds).

Method	Precision	Recall	F1	AHI MAE	AHI RMSE
This Study	0.8137±0.0332	0.8369±0.0470	0.8167±0.0052	1.3493±0.2165	1.8599±0.4112
Base U-Net	0.6484±0.0153	0.7321±0.0393	0.6762±0.0112	1.3220±0.2161	1.7966±0.4019

**Table 9:** Ablation on targeted training techniques (radar evaluation; mean ± std across folds).

Method	Precision	Recall	F1	AHI MAE	AHI RMSE
Full (with techniques)	0.8137±0.0332	0.8369±0.0470	0.8167±0.0052	1.3493±0.2165	1.8599±0.4112
Without techniques (all removed)	0.6844±0.0117	0.6464±0.0397	0.6427±0.0321	1.8835±0.1689	2.9255±0.4583

## Data Availability

The radar data presented in this study are available upon request from the corresponding author due to the protection of participants’ privacy.
